# Effect of continuous nursing on angina attack and quality of life in patients with coronary artery disease: A protocol for systematic review and meta-analysis

**DOI:** 10.1097/MD.0000000000024536

**Published:** 2021-02-05

**Authors:** Xiaohuan Zhou, Yamin Yuan, Zhanglin Wang, Ke Zhang, Weiwei Fan, Yawei Zhang, Pu Ma

**Affiliations:** a Pingdingshan University, Pingdingshan; b The First Affiliated Hospital of Zhengzhou University, Zhengzhou; c The First Affiliated Hospital of Henan University of Science and Technology, Luoyang, Henan Province, China.

**Keywords:** angina pectoris, continuous nursing, coronary artery disease, quality of life, systematic review

## Abstract

**Background:**

Coronary Artery Disease is an ischemic or necrotic heart disease caused by myocardial hypoxia caused by coronary artery stenosis or occlusion. The main symptoms are heart failure and recurrent angina pectoris. Continuous nursing refers to the nursing mode from in-hospital nursing to out-of-hospital nursing, including guiding patients’ follow-up treatment and lifestyle, which can effectively improve the quality of life in patients with Coronary Artery Disease and reduce the number of angina attacks. The study implemented in this program will systematically evaluate the efficacy and safety of continuous nursing intervention on an angina attack and quality of life in Coronary Artery Disease, and provide evidence-based basis for clinical application of continuous nursing intervention in Coronary Artery Disease.

**Method:**

The 2 researchers search the databases of China Knowledge Network, VP Information Chinese Journal Service Platform, PubMed, Embase, the Cochrane Library and Web of Science. From the establishment of the database in December 2020, all the randomized controlled trials on continuous nursing intervention for Coronary Artery Disease are collected. The relevant data are extracted and the quality is evaluated. meta-analysis is performed on the included literature using Stata15.0 software.

**Result:**

In this study, the efficacy and safety of continuous nursing intervention on Coronary Artery Disease are evaluated by Seattle angina questionnaire and other indicators.

**Conclusion:**

This study will provide reliable evidence for the clinical application of nursing intervention in Coronary Artery Disease.

**Ethics and dissemination:**

Private information from individuals will not be published. This systematic review also does not involve endangering participant rights. Ethical approval will not be required. The results may be published in a peer-reviewed journal or disseminated at relevant conferences.

**OSF Registration number:**

DOI 10.17605/OSF.IO/7QRKV.

## 1 Introduction

Coronary artery disease is full name of Coronary heart disease. Common causes include coronary atherosclerosis, coronary artery congenital malformation, coronary artery aneurysm, and so on.^[1]^ If atherosclerosis, thromboembolism or vasospasm occurs in the coronary artery, the lumen will be narrow or even blocked, and the blood flow will be blocked, so that the myocardium can not get adequate blood perfusion.^[2]^ Myocardial ischemia can easily lead to myocardial infarction or angina pectoris.^[3]^

At present, the main clinical treatment methods include: drug therapy,^[4]^ percutaneous coronary intervention,^[5]^ bypass surgery.^[6]^ The curative effect is good, which can effectively relieve the symptoms of angina pectoris and prevent the occurrence of dangerous events such as myocardial infarction.^[7]^ However, the direct cause of Coronary Artery Disease, atheromatous plaque can not be completely eradicated,^[8]^ easy to be affected by lifestyle. Most patients with Coronary Artery Disease have poor prognosis, delay and difficult to cure, and have higher mortality rate.^[9]^ The key to improve the prognosis of Coronary Artery Disease is to control the risk factors of atherosclerosis, so it is very important to take active and effective continuous nursing for patients with Coronary Artery Disease.^[10]^

Content of continuous care generally includes discharge plan, home guidance and follow-up investigation. Continuous nursing ensures that patients can still get professional care from hospital to home through the implementation of a variety of nursing measures.^[11]^ Continuous care can help patients establish a healthy lifestyle, maintain long-term family treatment, reduce the incidence of angina pectoris, and other complications,^[12]^ and improve the quality of life of patients.^[13]^

Although many studies have proved the efficacy of continuous nursing in the treatment of Coronary Artery Disease,^[12–19]^ there are still some problems, such as uneven trial quality, inconsistent safet,y and effectiveness, obvious individual differences, and so on. Therefore, we conducted a meta-analysis of currently available randomized controlled trials to explore the effect of continuous nursing on angina pectoris attack and quality of life in patients with Coronary Artery Disease, in order to provide evidence-based and scientific nursing intervention for patients with Coronary Artery Disease.

## 2 Method

### 2.1 Protocol register

This protocol of systematic review and meta-analysis has been drafted under the guidance of the preferred reporting items for systematic reviews and meta-analyses. It will be registered in the open science framework (OSF) (registration number: DOI 10.17605/OSF.IO/7QRKV).

### 2.2 Ethics

This study only uses the existing literature resources, does not belong to clinical research and does not require personal information, so it does not need the approval of the ethics committee.

### 2.3 Eligibility criteria

#### 2.3.1 Types of studies

We will include all randomized controlled trials of continuous nursing intervention for Coronary Artery Disease without date and language restrictions.

### 2.4 Research objects

Patients diagnosed as Coronary Artery Disease by clinical history, signs, symptoms, CT and electrocardiogram are in line with the diagnostic criteria in the clinical guidelines for the diagnosis and treatment of Coronary Artery Disease.^[20]^

#### 2.4.1 Interventions

The control group is treated with routine nursing during hospitalization, while the treatment group is treated with continuous nursing on the basis of routine nursing.

#### 2.4.2 Outcome indicators

##### 2.4.2.1 Main outcome: Seattle angina questionnaire

Secondary outcome: short form 36 questionnaire; China cardiovascular quality of life questionnaire; incidence of adverse reactions.

### 2.5 Exclusion criteria

(1) Repeatedly published research.
(2) For the study with incomplete data report, the original data can not be obtained after contacting the author.
(3) Study on the incompleteness of baseline data.
(4) Complicated with stroke, acute myocardial infarction, severe arrhythmia and other cardiovascular diseases.

### 2.6 Search strategy

From the establishment of the database in December 2020, all the Chinese and English literature on continuous nursing intervention for Coronary Artery Disease are collected. We will search eight databases by computer: PubMed, EMBASE, the Cochrane Library, Web of Science, China knowledge Network, VIP Chinese Journal Service platform, China Biomedical Database, Wanfang data knowledge Service platform. In addition, we will manually search relevant literature in Baidu academic and Google academic to supplement. With the keywords of “continuous care,” “Coronary Artery Disease,” “angina pectoris” and “quality of life,” the largest number of related literature are obtained by using Mesh and free words. Such as the retrieval strategy in PubMed in Table 1.

**Table 1:** Search strategy in PubMed database.

Number	Search terms
#1	Continuity of Patient Care[Mesh]
#2	Continuity of Patient Care[Title/Abstract]
#3	Continuum of Care[Title/Abstract]
#4	Continuity of Care[Title/Abstract]
#5	Care Continuum[Title/Abstract]
#6	Care Continuity[Title/Abstract]
#7	Continuous Nursing[Title/Abstract]
#8	#1 OR #2 OR #3 OR #4 OR #5 OR #6 OR #7
#9	Coronary Artery Disease[Mesh]
#10	Coronary Artery Disease[Title/Abstract]
#11	Coronary Arteriosclerosis[Title/Abstract]
#12	Coronary Atherosclerosis[Title/Abstract]
#13	Left Main Coronary Artery Disease[Title/Abstract]
#14	Left Main Disease[Title/Abstract]
#15	#9 OR #10 OR #11 OR #12 OR #13 OR #14
#16	#8 And #15

### 2.7 Data screening and extraction

**Figure 1:**
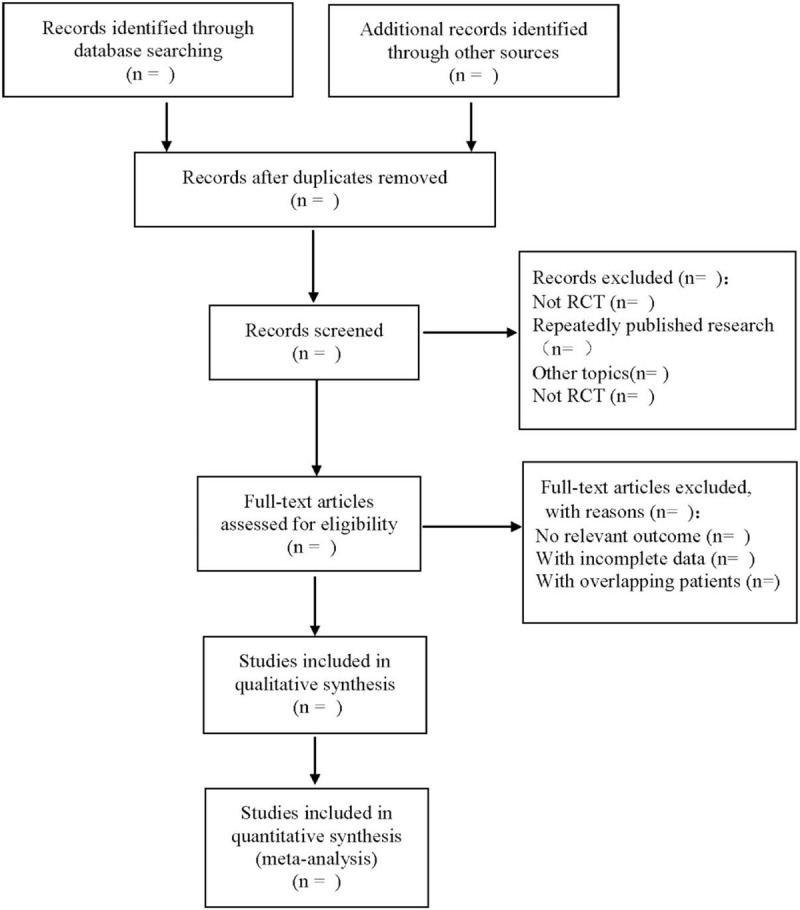
The process of literature screening.

We will use the Endnote X9 software to manage all the literature. Studies that meet the inclusion criteria will be independently searched by 2 researchers to systematically screen the titles. Abstracts and keywords of the articles searched, and exclude articles that do not meet the review criteria. The rest of the literature will be reviewed in full to exclude studies that do not conform to randomized controlled trials or studies with incomplete data. Finally, the relevant data of the literature in line with the research criteria are extracted from the original literature, including the author's name, article title, research site, publication year, study sample size, outcome indicators, detailed information of the test group and the control group, adverse events and so on. If the results of the 2 researchers are inconsistent, the differences can be resolved through discussion. If no agreement can be reached, the assistance of third-party researchers will be sought. The preferred reporting items for systematic reviews and meta-analyses flowchart is used to show the research selection process (Fig. 1).

### 2.8 Literature quality evaluation

Two researchers will use the bias risk assessment tool in the Cochrane manual to evaluate the methodological quality of randomized controlled trials. Each randomized controlled trial is evaluated with reference to the following conditions: a randomized method, allocation scheme concealment, blind method, data integrity, selective reporting, and other biases. Low-risk, unclear and high-risk judgments are given according to the performance of the included literature in the above evaluation items. After their completion, cross-check, if there are differences, they need to be discussed, and if no agreement can be reached, they will be agreed with third-party researchers.

### 2.9 Statistical analysis

#### 2.9.1 Data analysis and processing

We will use Stata15.0 software for statistical analysis. For binary variables, the risk ratio will be used to evaluate the efficacy of treatment. For continuous variables, if the measurement tools are the same, the weighted mean difference analysis is used, and if the measurement tools are different, the standardized mean difference analysis is used. The above results are expressed by 95% confidence interval. The forest map is used to show the results of meta-analysis. The results of *I*^2^ test and *P* test will be used as criteria for judging heterogeneity. When *P *≦* .1* and *I*^*2*^* ≥ 50%*, it is considered to have high heterogeneity. Therefore, we use random effects model to summarize the evaluation, and use subgroup analysis or meta regression to explore the sources of heterogeneity. If *P* *>* *.1* and *I*^*2*^ *<* *50%*, the homogeneity is considered to be better, and the fixed effect model is used.

#### 2.9.2 Dealing with missing data

If the data in the included study are missing or ambiguous, the author will be contacted by phone or email to provide more information. If the relevant data cannot be obtained, meta-analysis will be abandoned and descriptive analysis will be adopted.

#### 2.9.3 Subgroup analysis

According to the intervention methods, the subgroup analysis will be carried out for routine nursing and continuous nursing, the subgroup analysis will be carried out according to the age and sex of the patients, the subgroup analysis will be carried out according to the course of the disease, and the subgroup analysis will be carried out according to whether the patients had a history of operation. According to the clinical type, they are divided into five subgroups: Asymptomatic myocardial ischemia, angina pectoris, myocardial infarction, ischemic heart failure and sudden death.

#### 2.9.4 Sensitivity analysis

We will conduct a sensitivity analysis of the higher risk variables to test the quality and stability of the merger results.

#### 2.9.5 Assessment of reporting biases

When the number of articles included is more than 9, we will use Egger and Begg test to detect publication bias. If *P* < .05, it indicates that there is publication bias. If *P* > .05, there is no significant publication bias, and the result is reliable.

#### 2.9.6 Evidence quality evaluation

We will use the Grading of Recommendations Assessment, Development, and Evaluation) to grade the quality of the evidence. If the research has the risk of bias, inconsistency, indirectness, inaccurate information, and publication bias, the grade of evidence quality will be reduced as appropriate, and if it meets the conditions of larger effect, gradient dose-effect evidence and other conditions, the evidence quality will be upgraded as appropriate. Finally, the evidence quality is divided into four levels: high, medium, low and very low. The quality of evidence reflects the accuracy of the estimated value of the effect, and the high-quality evidence is often considered to be very close to the real value of the effect. But fundamentally speaking, evaluating the quality of evidence is still a subjective process.

## 3 Discussion

With the development of society and the change in life style, the prevalence and incidence of Coronary Artery Disease are increasing year by year.^[21]^ The occurrence and progress of Coronary Artery Disease is closely related to bad living habits.^[22]^ Hypertension, hyperglycemia, smoking, drinking, obesity, bad eating habits and so on will increase the risk of Coronary Artery Disease.^[23]^ In the concept of modern nursing management, continuous nursing has become an important part of high-efficiency and high-quality medical services.^[24]^ Providing continuous nursing for patients is an important factor to improve prognosis.^[25]^ It is also the embodiment and extension of nurses’ high-quality nursing service.^[26]^ As Coronary Artery Disease is greatly affected by daily life and the treatment cycle is long. It is necessary to carry out continuous care for patients with Coronary Artery Disease.^[15]^ Continuous nursing can effectively reduce the risk factors of patients exposed to Coronary Artery Disease and avoid recurrence and deterioration of the disease.^[27]^ Continuous nursing can also take personalized nursing measures according to the patient's condition, guide patients to use drugs reasonably, monitor the development of patients’ condition,^[18]^ and help patients develop a healthy lifestyle.^[28]^

At present, although many trials of continuous nursing intervention on Coronary Artery Disease have been reported, there is still a lack of systematic evaluation of continuous nursing intervention on Coronary Artery Disease. Therefore, it is necessary to make an objective evaluation of continuous nursing intervention in Coronary Artery Disease based on evidence-based medicine and promote continuous nursing.

This study also has some limitations, the inherent defects in the existing published literature may affect the results; the patients in the study may not represent the overall population; there may be some methodological heterogeneity and clinical heterogeneity, if too significant, the reliability of the results remains to be studied.

## Author contributions

**Data curation:** Xiaohuan Zhou, Yamin Yuan.

**Funding acquisition:** Xiaohuan Zhou.

**Resources:** Zhanglin Wang, Ke Zhang.

**Software:** Yawei Zhang, Pu Ma.

**Supervision:** Zhanglin Wang, Weiwei Fan.

**Writing – original draft:** Xiaohuan Zhou, Yamin Yuan.

**Writing – review & editing:** Xiaohuan Zhou.
